# A novel linker-immunodominant site (LIS) vaccine targeting the SARS-CoV-2 spike protein protects against severe COVID-19 in Syrian hamsters

**DOI:** 10.1080/22221751.2021.1921621

**Published:** 2021-05-09

**Authors:** Bao-Zhong Zhang, Xiaolei Wang, Shuofeng Yuan, Wenjun Li, Ying Dou, Vincent Kwok-Man Poon, Chris Chung-Sing Chan, Jian-Piao Cai, Kenn KaHeng Chik, Kaiming Tang, Chris Chun-Yiu Chan, Ye-Fan Hu, Jing-Chu Hu, Smaranda Ruxandra Badea, Hua-Rui Gong, Xuansheng Lin, Hin Chu, Xuechen Li, Kelvin Kai-Wang To, Li Liu, Zhiwei Chen, Ivan Fan-Ngai Hung, Kwok Yung Yuen, Jasper Fuk-Woo Chan, Jian-Dong Huang

**Affiliations:** a CAS Key Laboratory of Quantitative Engineering Biology, Shenzhen Institute of Synthetic Biology, Shenzhen Institutes of Advanced Technology, Chinese Academy of Sciences, Shenzhen, People’s Republic of China; b School of Biomedical Sciences, Li Ka Shing Faculty of Medicine, The University of Hong Kong, Pokfulam, People’s Republic of China; c State Key Laboratory of Emerging Infectious Diseases, Li Ka Shing Faculty of Medicine, The University of Hong Kong, Pokfulam, People’s Republic of China; d Department of Microbiology, Li Ka Shing Faculty of Medicine, The University of Hong Kong, Pokfulam, People’s Republic of China; e Department of Chemistry, Faculty of Science, The University of Hong Kong, Pokfulam, People's Republic of China; f AIDS Institute, Li Ka Shing Faculty of Medicine, The University of Hong Kong, People’s Republic of China; g Department of Medicine, Li Ka Shing Faculty of Medicine, The University of Hong Kong, Pokfulam, People’s Republic of China

**Keywords:** COVID-19, SARS-CoV-2, linker-immunodominant site, spike protein, vaccine

## Abstract

The Coronavirus Disease 2019 (COVID-19) pandemic is unlikely to abate until sufficient herd immunity is built up by either natural infection or vaccination. We previously identified ten linear immunodominant sites on the SARS-CoV-2 spike protein of which four are located within the RBD. Therefore, we designed two linkerimmunodominant site (LIS) vaccine candidates which are composed of four immunodominant sites within the RBD (RBD-ID) or all the 10 immunodominant sites within the whole spike (S-ID). They were administered by subcutaneous injection and were tested for immunogenicity and *in vivo* protective efficacy in a hamster model for COVID-19. We showed that the S-ID vaccine induced significantly better neutralizing antibody response than RBD-ID and alum control. As expected, hamsters vaccinated by S-ID had significantly less body weight loss, lung viral load, and histopathological changes of pneumonia. The S-ID has the potential to be an effective vaccine for protection against COVID-19.

## Introduction

The Coronavirus Disease 2019 (COVID-19) pandemic caused by severe acute respiratory syndrome coronavirus 2 (SARS-CoV-2) has resulted in significant mortality, morbidity, and socioeconomic disruptions globally [1]. As of 15 December 2020, SARS-CoV-2 is highly efficient in person-to-person transmission and has caused more than 70 million cases with nearly 1.6 million deaths [2] [https://www.who.int/publications/m/item/weekly-epidemiological-update---15december-2020]. Symptomatic COVID-19 is characterized by systemic symptoms such as fever, myalgia, and lethargy, respiratory symptoms, and occasionally extrapulmonary manifestations such as diarrhea, anosmia, and ageusia [3]. It has been estimated that at least about 20% of COVID-19 patients are asymptomatic, which makes the control of the pandemic particularly difficult [2,4]. An effective and vaccine for COVID-19 is an essential countermeasure to halt the continuous dissemination of SARS-CoV-2 [5].

SARS-CoV-2 is a lineage B betacoronavirus which shares high sequence homology with SARS-CoV [6,7]. Like SARS-CoV, SARS-CoV-2 employs its highly glycosylated spike (S) protein to enter to host cells. The receptor binding domain (RBD) at the S1 subunit of SARS-CoV-2 S protein interacts with the host surface receptor angiotensinconverting enzyme II (ACE2) to trigger virus-host cell membrane fusion [8]. Vaccine candidates developed using various platforms are being evaluated in different phases of clinical trials [9–13]. As of mid-December 2020, a number of COVID-19 vaccines, namely, the mRNA vaccine mRNA-1273 (Moderna), the inactivated vaccine CoronaVac (SinoVac), and the chimpanzee adenoviral-vectored vaccine ChAdOx1 (Oxford/AstraZeneca), have been approved for emergency use in different parts of the world. We previously showed that patients can mount neutralizing antibody response against the receptor binding domain and N terminal domain of the S protein [14].

The persistence of the COVID-19 pandemic allows SARS-CoV-2 to continuously adapt to human with accumulation of immunologically relevant mutations in the population [15,16]. Antigenic drift has been documented among coronaviruses [17–20]. For example, the D480A/G mutation in the RBD of SARS-CoV-1 became the dominant variant during the SARS outbreak in 2003 [20]. In the case of SARS-CoV-2, the D614G mutation in S protein is now found in the majority of the virus strains. Understanding B cell and antibody immunodominance is important for rational design of vaccines that can induce neutralizing antibodies against pathogens with antigenically variable strains [21,22]. We recently conducted a detailed analysis of the serological data of COVID19 patients and identified ten linear immunodominant (ID) sites on the SARS-CoV-2 S protein [23]. Some of these ID sites coincide with conformational epitopes identified by using S protein-specific monoclonal antibodies. We therefore asked whether these ID sites alone without the interlinking non-ID sites could serve as a novel vaccine that provides sufficient protective immunity against SARS-CoV-2.

In this study, we designed two linker-immunodominant sites (LIS) candidate vaccines composed of four or ten ID sites on the S protein. We demonstrated that both candidate vaccines could induce robust humoral and cellular response in mice and one of the candidates provided protection against severe disease in the golden Syrian hamster model for COVID-19.

## Materials and methods

### Cell line and viruses

SARS-CoV-2 (strain HKU-001a, GenBank accession number: MT230904) was propagated as previously described [24]. Stable cells of HEK293-hACE2 were prepared as previously described [25]. All cells were maintained in DMEM supplemented with 10% heat-inactivated fetal bovine serum (FBS), 50 U/ml penicillin and 50 μg/ml streptomycin. SARS-CoV-2 pseudoviral particles [replication-deficient murine leukaemia virus (MLV) pseudotyped with the SARS-CoV-2 spike protein] were purchased from eEnzyme (cat no. SCV2-PsV-001). Cells were confirmed to be free of mycoplasma contamination by PlasmoTest (InvivoGen). All experiments involving live SARS-CoV-2 were conducted according to the approved standard operating procedures of the Biosafety Level 3 facilities at The University of Hong Kong (HKU) [26].

## RBD-ID and S-ID polypeptide purification

The RBD-ID and S-ID DNA sequence was synthesized (BGI) and cloned into pET28a, resulting in p-4ID and p-10ID plasmid, respectively. To facilitate the purification, a hexa-His tag was added to the C-terminal of RBD-ID and S-ID. P-4ID and p-10ID were subsequently transformed into *E. coli* BL21(DE) strain. Bacterial pellet from 1L of bacterial culture were harvested by centrifugation. Followed by sonication, the pellets were resuspended in binding buffer (Tris-HCl pH 8.0, 20 mM imidazole). The soluble cell extracts were collected and filtered using a 0.22 μm membrane after centrifugation at 15,000 × g for 30 min at 4°C. The filtrates were then loaded on a nickel-NTA Sepharose column (GE Healthcare). The bound proteins were eventually eluted with elution buffer (Tris-HCl pH 8.0, 250 mM imidazole). The purified proteins were identified using SDS-PAGE analysis and subject to endotoxin removal afterwards (Pierce).

## Active immunization

RBD-ID and S-ID vaccine formulated with aluminum hydroxide gel adjuvant (Alum; 1 mg/ml) were injected subcutaneously (s.c.) into BALB/c mice (10 per group, 25 µg/animal/injection) and golden Syrian hamsters (5 per group). The animals were subjected to booster vaccinations every 3 weeks (administered twice). Animals of the mock-immunized group received an equal amount of Alum in a mixture with PBS. The antibody titers in the serum samples collected from animals were documented by enzyme-linked immunosorbent assay (ELISA) after each booster vaccination as described elsewhere [23].

## ELISPOT assay

Mice were sacrificed 5 days after the second booster vaccination (s.c.). After euthanasia, spleens were collected and single suspensions of splenocytes were obtained. Interferon gamma (IFN-)-producing and interleukin 17A (IL-17A)-producing splenocytes from vaccinated or control mice were analysed using a cytokine-specific enzyme linked immunospot (ELISPOT) assay (R&D Systems, USA) as described by the manufacturer. Briefly, splenocytes isolated from immunized mice were plated at a concentration of 1 × 105 cells/well and induced with antigens (0.2 µg/well) in triplicate and incubated for 20 h at 37°C. Ionomycin (Sigma, USA) (1 µg/ml) and phorbol myristate acetate (PMA; Sigma) (50 ng/ml) were used as positive controls. Splenocytes from unstimulated, immunized mice and RPMI 1640-treated splenocytes were used as negative controls. After the cells were decanted, biotinylated primary monoclonal antibodies were added to each well and the plates were incubated for 1 h at 37°C. The plates were incubated with streptavidin-horseradish peroxidase (HRP) conjugate for 1 h at 37°C and subjected to colour development with TMB solution. Finally, the spots were enumerated using an Immunospot analyzer.

## Pseudovirus-based virus neutralization test

Pseudovirus-based virus neutralization test (pVNT) was performed as described previously, with modifications [25]. In brief, pVNT was performed in HEK293-hACE2 stable cells. Murine leukemia virus–based SARS-CoV-2 spike-pseudotyped particles (Wuhan-Hu-1 strain) were purchased from eEnzyme (catalog number SCV2-PsV-001). Sera were serially diluted with phosphate-buffered saline (PBS) and co-incubated with the equal volume of pseudovirus (1:10 dilution of the stock) for 30 min before adding to the cells. After incubation at 37°C for 36 h, firefly luciferase activity indicating pseudovirus entry was determined using the Luciferase Assay System (Promega, catalog number E1501). Serum samples of five randomly selected COVID convalescent patients from 39 patients with COVID-19 between Jan 26, 2020, and March 18, 2020 were tested for neutralizing antibody titers. The patients provided written informed consent under UW 13-372, UW 13-265, and UW 19-470. The antiRBD immune serum was kindly provided by Prof. Kwok-yung Yuen's group.

## Viral challenge experiment in golden Syrian hamsters

Male and female Syrian hamsters, aged 4–6 weeks old, were obtained from the Chinese University of Hong Kong Laboratory Animal Service Centre through the HKU Centre for Comparative Medicine Research. All the animal experimental procedures were approved by the Committee on the Use of Live Animals in Teaching and Research of HKU. Briefly, the hamsters were with subcutaneous two injections of RBD-ID, S-ID, or aluminum hydroxide gel (control) at day 0 and day 21, kept in Biosafety Level 2 housing and given access to standard pellet feed and water ad libitum, as previously described [25]. Each hamster was anesthetized with intraperitoneal ketamine (200 mg/kg) and xylazine (10 mg/kg) and then intranasally inoculated with 10^5^ plaque forming units (pfu) of SARS-CoV-2 in 100 µl DMEM. The hamsters were monitored twice daily for clinical signs of disease until 4 dpi when they were sacrificed for virological and histopathological analyses. Viral load and infectious virus titers in the lung tissue homogenates were quantified by qRT–PCR and plaque assay as previously described [26]. Histopathological analysis and immunofluorescence staining were performed as previously described [26]. The histological scores were determined as we described previously [27].

## Statistical analysis

All data were analysed in GraphPad prism 7.0 software (GraphPad Software Inc., CA, US). Differences between two groups were compared by unpaired Student's t-test.

Differences among three or more groups were compared by one-way ANOVA. Twotailed *p* value < 0.05 was considered statistically significant.

## Supplementary Material

COVID19_LIS_Vaccine-_SM_2021_03_31_editable.docx

## Figures and Tables

**Figure 1.**
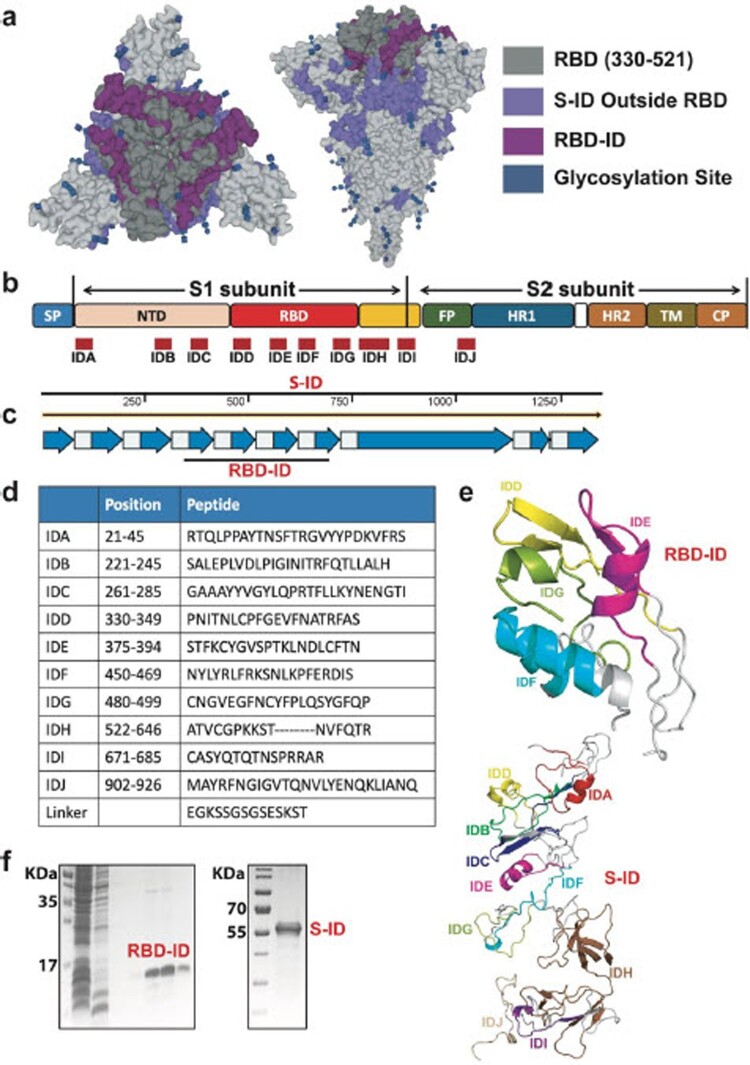
Design of linker-immunodominant site (LIS) COVID-19 vaccine candidates. (a) Structural representation and (b) schematic view of the immunodominant sites in the SARS-CoV-2 spike protein. Abbreviations: SP, signal peptide; NTD, N-terminal domain; RBD, receptor-binding domain; FP, fusion peptide; HR, heptad repeat; TM, transmembrane domain; CP, cytoplasmic domain. (c) Schematic depiction of the RBD-ID and S-ID gene sequences. (d) The amino acid sequences of the 10 immunodominant sites. (e) Representation of the predicted RBD-ID and S-ID structures. (f) SDS-PAGE analysis of the recombinant RBD-ID (14 kDa) and S-ID (48 kDa).

**Figure 2.**
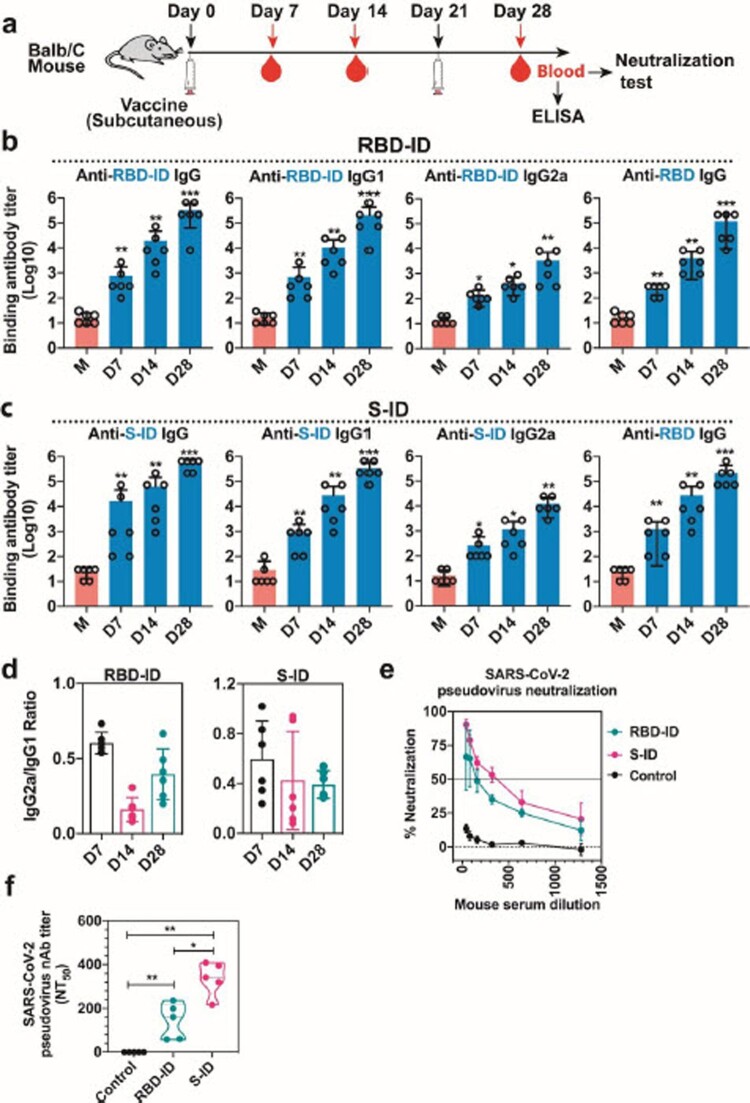
Humoral immune responses in RBD-ID-iummunized or S-IDimmunized BALB/c mice. The BALB/c mice were immunized subcutaneously with 25 μg recombinant RBD-ID (*n* = 6), S-ID (*n* = 6), or aluminum hydroxide gel (control, *n* = 6) per mouse, and boosted with an equivalent dose 21 days later. Serum samples were collected from the mice at days 7, 14, 21, and 28 after the initial immunization and tested for binding antibody and neutralizing antibody titers. (a) Schedule of immunization and sample collection. (b) IgG subclass binding antibodies against RBD-ID or RBD in mice immunized with RBD-ID detected by ELISA. Data are presented as mean ± SD. Statistical significance was calculated using a two-way ANOVA with multiple comparisons tests (*p < 0.05, **p < 0.01, ***p < 0.001). (c) IgG subclass binding antibodies against S-ID or RBD in mice immunized with SID detected by ELISA. Data are presented as mean ± SD. Statistical significance was calculated using a two-way ANOVA with multiple comparisons tests (*p < 0.05, **p < 0.01, ***p < 0.001). (d) Endpoint titer ratios of IgG2a/c to IgG1. Data are shown as mean ± SD. (e) % neutralization and (f) 50% neutralizing antibody titer achieved by RBD-ID, S-ID, or control in the sera of immunized mice. Neutralization was determined using VSVbased SARS-CoV-2 pseudovirus. The dashed line indicated the detection limit or and the solid line indicated 50% neutralization. Data are shown as mean ± SD.

**Figure 3.**
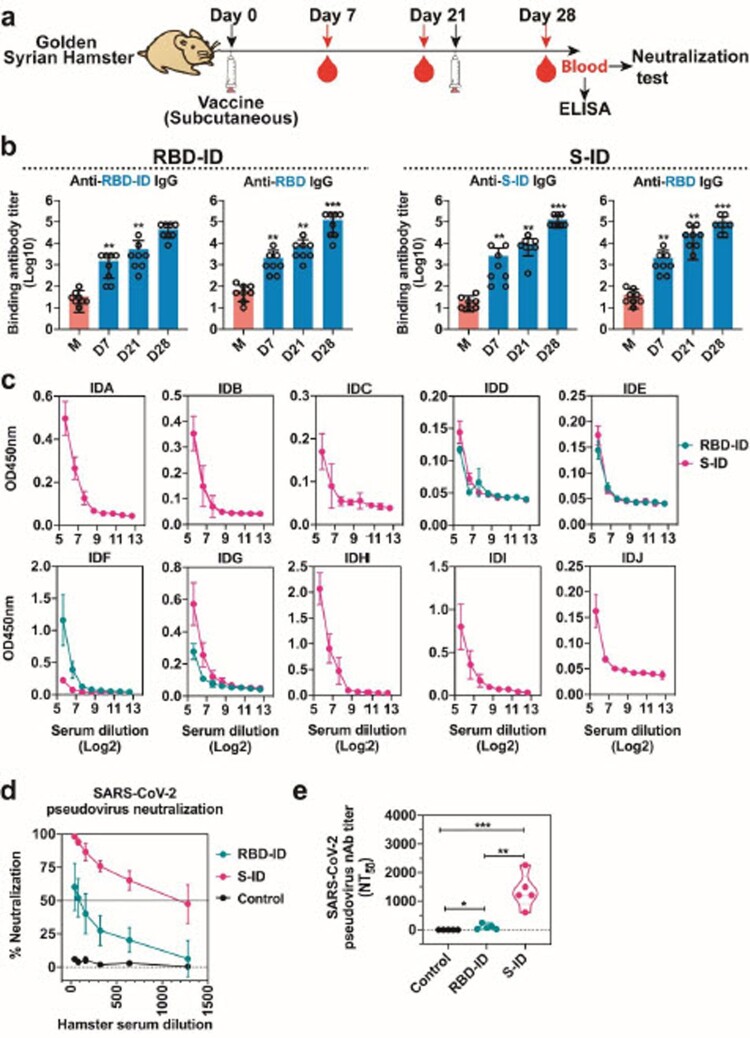
Humoral immune responses in immunized golden Syrian hamsters. The hamsters were immunized subcutaneously with 25 μg recombinant RBD-ID (*n* = 5), SID (*n* = 5), or aluminum hydroxide gel (control, *n* = 5) per hamster, and boosted with an equivalent dose 21 days later. Serum samples were collected from the mice at days 7, 21, and 28 after the initial immunization and tested for binding antibody and neutralizing antibody titers. (a) Schedule of immunization and sample collection. (b) IgG subclass binding antibodies against RBD-ID, S-ID, or RBD in hamsters immunized with RBD-ID (left) or S-ID (right) detected by ELISA. Data are presented as mean ± SD. Statistical significance was calculated using a two-way ANOVA with multiple comparisons tests (*p < 0.05, **p < 0.01, ***p < 0.001). (c) Immunodominant site-specific antibodies in the hamsters. (d) % neutralization and (e) 50% neutralizing antibody titer achieved by RBD-ID, SID, or control in the sera of immunized hamsters. Neutralization was determined using VSV-based SARS-CoV-2 pseudovirus. The dashed line indicated the detection limit or and the solid line indicated 50% neutralization. Data are shown as mean ± SD.

**Figure 4.**
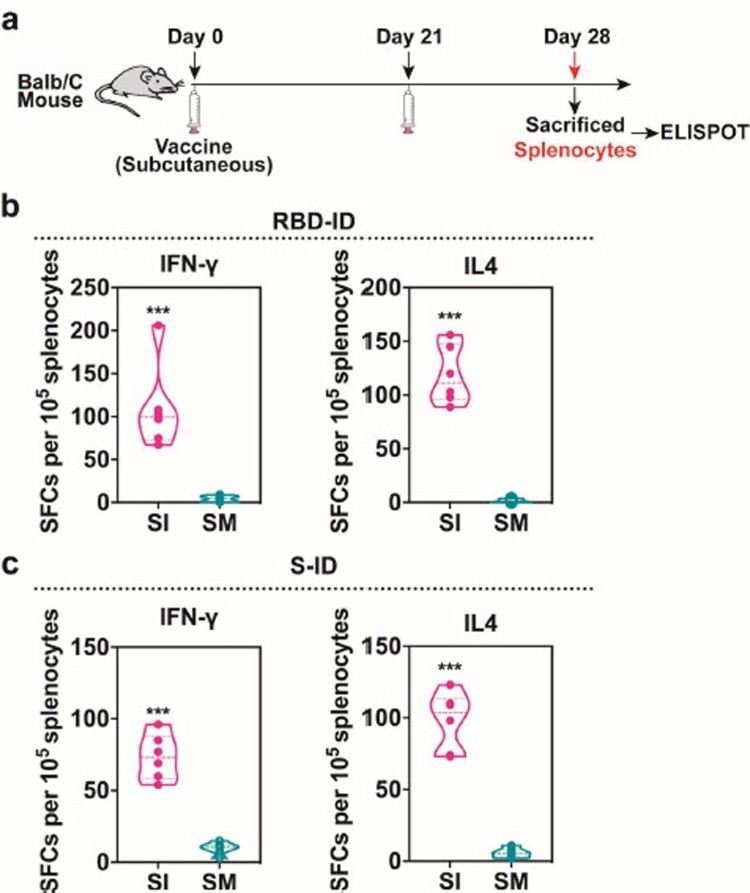
Cellular immune responses in RBD-ID-iummunized or S-ID-immunized BALB/c mice. The BALB/c mice were immunized subcutaneously with 25 μg recombinant RBD-ID (*n* = 6), S-ID (*n* = 6), or aluminum hydroxide gel (control, *n* = 6) per mouse, and boosted with an equivalent dose 21 days later. Splenocytes were collected at days 28 after the initial immunization and tested for T cell response. (a) Schedule of immunization and sample collection. (b) ELISPOT assay for IFN-γ and IL-4 in the splenocytes of the mice immunized with RBD-ID or (c) S-ID. Data are shown as violin plot with all points. Unpaired two-tailed Student's t-test, **p < 0.01. Abbreviations: SI, immunized mice stimulated with RBDID or S-ID; SM, mock-immunized mice stimulated with RBD-ID or S-ID.

**Figure 5.**
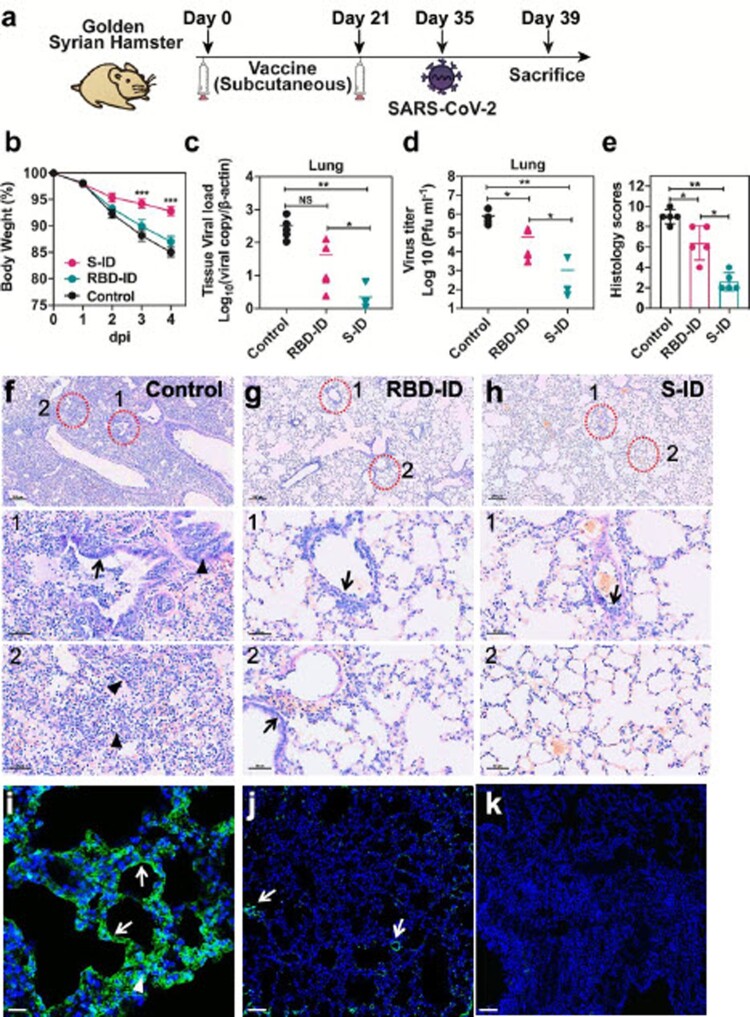
S-ID immunization protects golden Syrian hamsters from severe SARSCoV-2 infection. At 35 days after the initial immunization, hamsters (*n* = 5 per group) were intranasally inoculated with 10^5^ plaque forming units (pfu) of SARS-CoV-2 in 100 µl DMEM. The hamsters were sacrificed at 4 days after virus challenge for tissue collection for viral loads, virus titer, and lung pathology evaluation. (a) Schedule of immunization, virus challenge, and tissue collection. (b) Body weight changes of the hamsters. Error bars denote SD. (c) The viral loads and (d) virus titers in the lungs of the hamsters at 4 days after virus challenge determined by quantitative RT-PCR and median tissue culture infective dose (TCID_50_) assay, respectively. Data are presented as mean ± SD. Statistical significance was calculated using a two-way ANOVA with multiple comparisons tests (*p < 0.05, **p < 0.01, ***p < 0.001). (e) Histological scores indicating the lung histopathological severity in each group. Data are presented as mean ± SD of five randomly selected slides from each group. Unpaired two-tailed Student's t-test, **p < 0.01 when compared with the placebo group. (f) to (h) Representative images of the H&E-stained lung tissues of (f) mockimmunized, (g) RBD-ID-immunized, or (h) S-ID-immunized hamsters at 4 days after SARS-CoV-2 challenge. The mock-immunized hamsters showed diffuse lung tissue consolidation with alveolar infiltration and exudation, bronchiolar epithelium cell death (arrows), and peribronchiolar mononuclear cell infiltration (arrowheads). These changes were less severe in the RBD-ID-immunized hamsters and the least severe in the S-ID-immunized hamsters. (i) to (k) Representative images of the immunofluorescent staining using SARS-CoV2 nucleocapsid protein of the lung tissues of (i) mock-immunized, (j) RBD-IDimmunized, or (k) S-ID-immunized hamsters at 4 days after SARS-CoV-2 challenge. The mock-immunized hamsters showed abundant SARS-CoV-2 nucleocapsid protein expression diffusely distributed in the alveola (white arrowheads) and in the bronchiolar epithelial cells (white arrows). Scale bar, 100 μm. The RBD-ID-immunized hamsters showed markedly less SARS-CoV-2 nucleocapsid protein expression and the S-IDimmunized hamsters exhibited almost no detectable SARS-CoV-2 nucleocapsid protein expression.

## Data Availability

All data is available upon request. Unique materials used in this study are available from the corresponding author by Material Transfer Agreement.

## Results

### Generation of LIS vaccine candidates

In a recent study, we identified ten linear immunodominant (ID) sites on the SARSCoV-2 S protein located at 21–45 (IDA), 221–245 (IDB), 261–285 (IDC), 330–349 (IDD), 375–394 (IDE), 450–469 (IDF), 480–499 (IDG), 522–646 (IDH), 671–685 (IDI), and 902–926 (IDJ) (Figure 1(a–d) and Fig.S1). Four of these ID sites, namely, IDD, IDE, IDF, and IDG, are located within the receptor-binding domain (RBD). We designed two linker-immunodominant site (LIS) vaccine candidates composed of these four ID sites within the RBD (RBD-ID) or the complete ten ID sites (S-ID) (Figure 1(e)). The peptide sequences of the ID sites are aligned with peptide linkers, expressed, and purified by using affinity chromatography. SDS-PAGE analysis of recombinant RBDID (14 kDa) and S-ID (48 kDa) was performed to confirm the purity (Figure 1(f)).

## Humoral responses elicited by immunization with RBD-ID or S-ID

The ability to elicit a robust humoral response is required for an effective coronavirus vaccine. To this end, we first immunized BALB/c mice with either one of our two vaccine candidates on day 0 and day 28 by subcutaneous injection (Figure 2(a)). Enzymelinked immunosorbent assay (ELISA) was performed on the mouse sera to analyse the humoral immune response after each injection. Immunization of both RBD-ID and SID induced RBD-specific IgG responses (Figure 2(b,c)). We also compared the levels of RBD-specific IgG1 and IgG2a in the mouse sera to evaluate the balance of Th1 and Th2-biased immunity. Both RBD-ID and S-ID induced IgG1 and IgG2a subclass antibodies (Figure 2(d)). The IgG subclass profile indicated that Th2-biased antibodies was elicited in both RBD-ID-immunized and S-ID-immunized mice. Pseudovirus neutralizing activity was induced by immunization with either RBD-ID or S-ID, with S-ID immunization having a higher NT_50_ (Figure 2(e,f), Fig.S2).

Next, immune sera from golden Syrian hamsters were tested for immunogenicity and neutralizing activity against SARS-CoV-2 (Figure 3(a)). As shown in Figure 3(b), high RBDspecific and antigen-specific IgG titers were detected in the sera of hamsters immunized with either RBD-ID or S-ID. Furthermore, both RBD-ID and S-ID could elicit ID sitespecific antibodies in the hamsters (Figure 3(c)). Immune sera from the hamsters effectively blocked infection by SARS-CoV-2 pseudovirus (Figure 3(d–e), Fig S2). In particular, 1:40 dilution of sera from S-ID-immunized hamsters could almost completely protect HEK293-hACE2 stable cells from SARS-CoV-2 pseudovirus infection (Figure 3(d)). Accordingly, the mean of NT_50_ of S-ID (1355) was about 14-fold higher than that of RBD-ID (99.45) (Figure 3(e)).

## Cellular immune responses induced by immunization with RBD-ID or S-ID

In addition to humoral response, we further investigated whether a SARS-CoV-2specific cellular immune response was induced by immunization of RBD-ID or S-ID in BALB/c mice (Figure 4(a)). As shown in Figure 4(b,c), enzyme-linked immunosorbent spot (ELISPOT) assay showed that the number of IFN-γ-producing splenocytes was much higher in the mice immunized with either RBD-ID or S-ID. Accordingly, the number of IL-4-producing splenocytes in the immunized mice were significantly higher than that of the control mice.

## Evaluation of protective efficacy elicited by RBD-ID or S-ID against SARS-CoV-2 infection in golden Syrian hamsters

As immunization of RBD-ID and S-ID could elicit both high-titer neutralizing antibodies and robust T cell response, we proceeded to study whether the two LIS vaccine candidates could block or reduce the infection in golden Syrian hamsters inoculated with live SARS-CoV-2. The hamsters were immunized with two subcutaneous injections (25 µg per dose) of either RBD-ID or S-ID on day 0 and day 21 and then challenged with SARS-CoV-2 14 days after the final injection (Figure 5(a)). Aluminum hydroxide gel alone was used for the control hamsters. As shown in Figure 5(b), the control hamsters developed weight loss of about 15% at 4 dpi, whereas the hamsters immunized with S-ID had minimal weight loss of about 8% at 4 dpi. Corroborating with the weight loss results, the viral loads, viral titer (Figure 5(c,d)), and the histology scores (Figure 5(e)) of the hamsters immunized with S-ID were the lowest among the three groups and were significantly lower than those of the control hamsters and the hamsters immunized with RBD-ID.

Hematoxylin and eosin stain (H&E stain) analysis of the control hamsters’ lung tissues showed severe mononuclear inflammatory cell infiltration and distorted alveolar structures with diffuse loss of alveolar space (Figure 5(f)). The lung tissues of the hamsters immunized with RBD-ID displayed milder histopathological changes with moderate mononuclear inflammatory cell infiltration but largely preserved alveolar space (Figure 5(g)). In contrast, the lung tissues of the hamsters immunized with S-ID exhibited minimal histopathological changes with little inflammatory cell infiltration largely preserved alveolar structures (Figure 5(h)). In line with the H&E stain findings, immunostaining assay detected abundant, moderate, and minimal SARS-CoV-2 nucleocapsid protein expression in the lung tissues of the control hamsters, hamsters immunized with RBD-ID, and hamsters immunized with S-ID, respectively (Figure 5(i–k)).

## Discussion

The rapid spread of COVID-19 caused by SARS-CoV-2 has led to global panic and urgent need of effective vaccination strategies. The S protein of coronaviruses is known to be highly immunogenic and may serve as a vaccine target [28,29]. We recently mined the immunogenic epitopes on S protein of SARS-CoV-2 by analysing sera samples of COVID-19 patients and identified ten linear ID sites, including four which reside in the RBD [23]. Based on these findings, we developed two novel candidate LIS vaccines by incorporating the ten ID sites into single polypeptides. We then thoroughly evaluated the humoral and cellular immunity they induced in BALB/c mice as well as their protection against SARS-CoV-2 infection in our established golden Syrian hamster model. While both RBD-ID and S-ID induced high titers of binding antibodies and cellular immune response, only S-ID induced robust neutralizing antibody response in mice. Importantly, our data showed that immunization with S-ID which encompasses all ten ID sites could significantly improve the clinical, virological, and histopathological parameters in hamsters. RBD-ID, which only contains the four ID sites within the RBD, did not confer as much protection as S-ID in vivo. Immunization of hamsters with RBD-ID elicited antibodies specific for all four ID sites. However, pseudovirus neutralization assay showed that the anti-SARS-CoV-2 neutralizing activity of RBD-ID immune sera was much lower than that of S-ID. The mean serum neutralizing antibody titers of S-ID-immunized mice and hamsters were 2.12 and 13.9 folds higher than those of RBD-ID-immunized mice and hamsters, respectively. Furthermore, we compared the neutralizing antibody titers of serum samples from COVID-19 convalescent patients and anti-RBD serum with that of S-ID immune serum.

The serum neutralizing antibody titers of S-ID immunized hamsters were comparable with those of convalescent patients and RBD immunized hamsters (Fig. S2). These results provided the first in vitro and in vivo evidence that LIS vaccine which includes the appropriate ID sites can induce robust humoral and cellular immune response against SARS-CoV-2.

A recent study reported a map of the major RBD antigenic sites through structural studies of monoclonal antibodies (mAbs) derived from COVID-19 patients [30]. Interestingly, despite that the ID sites E (375–394), F (450–469), and G (480–499) overlapped with several binding sites recognized by the reported neutralizing mAbs, RBD-ID containing these ID sites did not confer strong protection against SARS-CoV2 infection in vivo. This might be related to the intrinsic limitation of RBD-ID which used artificial peptide linkers to link the four linear ID sites and thus might induce a less potent neutralizing antibody response than the conformational epitopes recognized by the monoclonal antibodies. The stronger protection conferred by S-ID, as compared with RBD-ID, highlights the importance of the six ID sites located outside the RBD in the S1 subunit of the SARS-CoV-2 S protein. The immunogenicity of these ID sites is further confirmed by in vitro neutralization assay. In contrast with the abundant information available for identification of epitopes within the RBD [30–40], our knowledge on the interactions between the epitopes outside of the RBD and SARSCoV-2-specific antibodies remains limited. A linear epitope (562–580) located within ID site H (522–646) of S-ID was recently found to induce neutralizing antibodies in COVID-19 patients in two different studies [41,42]. ID site I (671–685) is located within the S1/S2 cleavage site that is essential for virus-host membrane fusion [43], and thus antibodies elicited by ID site I may be able to block the membrane fusion process. Considering the proximity of some of these six extra-RBD ID sites to RBD, antibodies induced by these ID sites might sterically hinder binding of RBD to the ACE2 receptor. Taken together, our data demonstrated that the inclusion of extra-RBD ID sites without the interlinking non-ID sites in candidate vaccines can contribute to a more potent neutralizing antibody response than vaccines which only contain RBD ID sites.

Overall, our data clearly demonstrated that the S-ID containing both intra- and extraRBD ID sites robustly induced the host humoral and cellular immune responses, resulting in significantly improved disease outcome in vivo. Our findings in this study provided a new platform for the rational design of vaccines against SARS-CoV-2 and possibly other emerging viruses.
