# Supplementary material for: A novel linker-immunodominant site (LIS) vaccine targeting the SARS-CoV-2 spike protein protects against severe COVID-19 in Syrian hamsters
Source: Emerg Microbes Infect. 2021 May 9;10(1):874–84. doi: 10.1080/22221751.2021.1921621 (PMC8118541; doi:10.1080/22221751.2021.1921621)
Supplement: COVID19_LIS_Vaccine-_SM_2021_03_31_editable.docx [file TEMI_A_1921621_SM0841.docx]

1. **A novel linker-immunodominant site (LIS) vaccine targeting the SARS-CoV-2**
2. **spike protein protects against severe COVID-19 in Syrian hamsters**
3. **Supplementary Figures**
4.
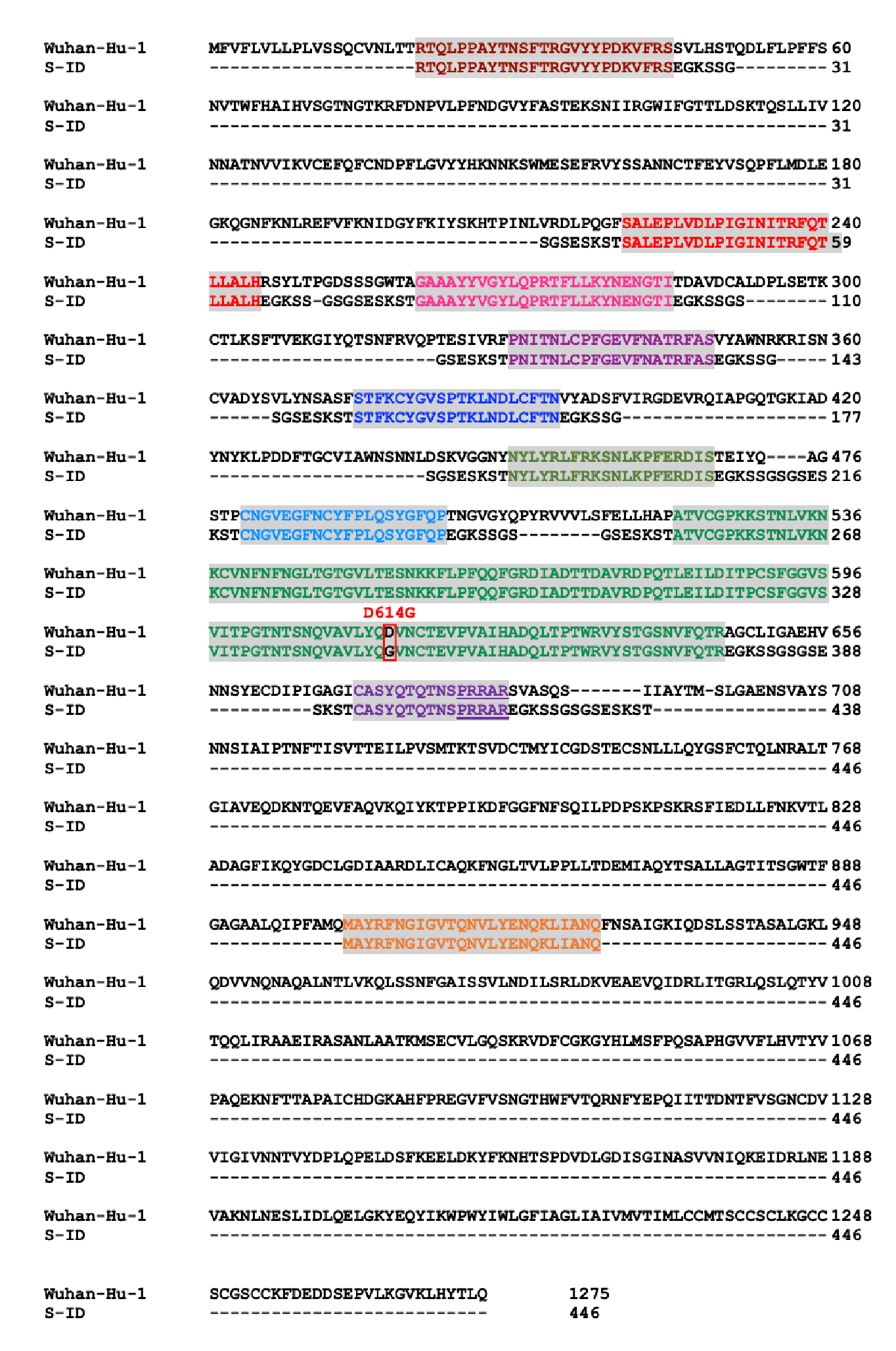

5. **Figure S1:** Amino acid sequence alignment of the full S protein of SARS-CoV-2 6 isolates used in this study, Related to Figures 1 ID are shown as colours.

7

8


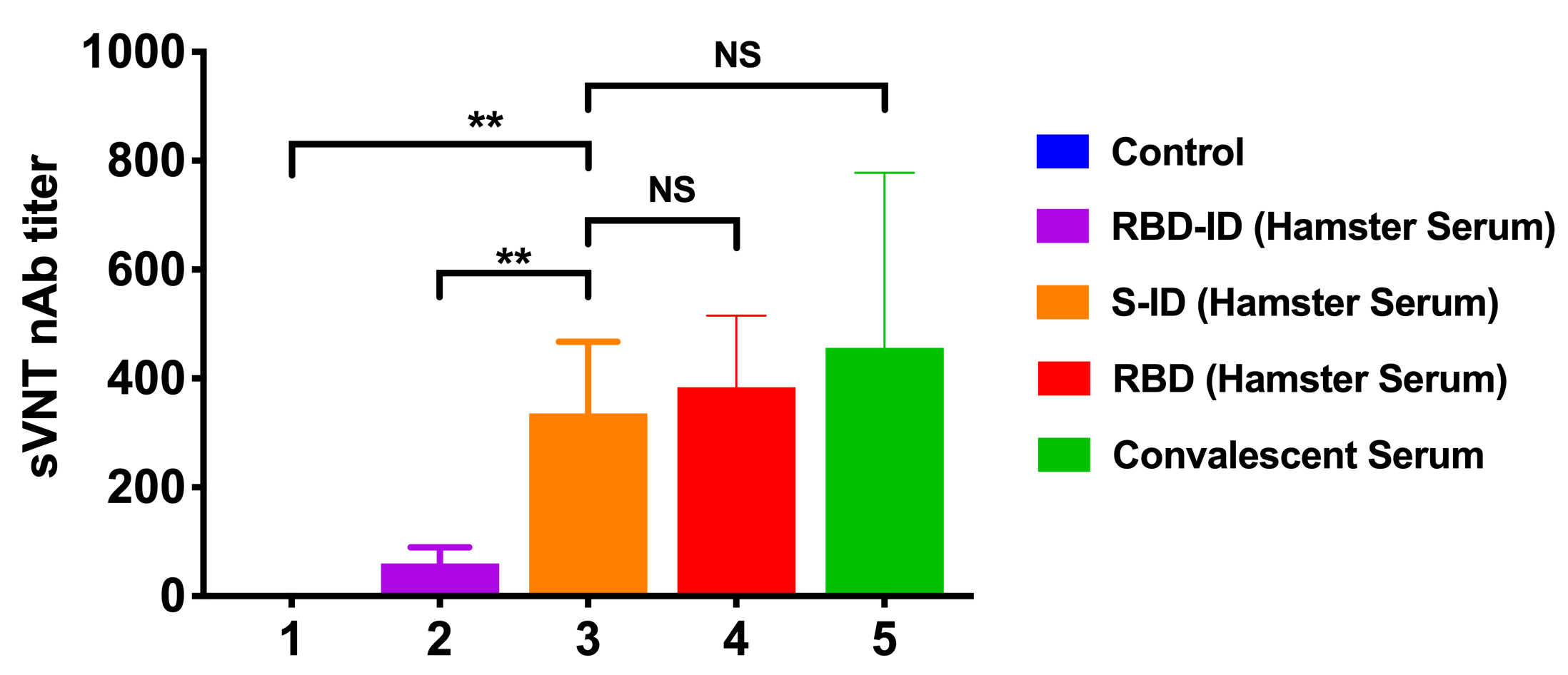


**Figure S2.** Surrogate Virus Neutralization Test (sVNT) was determined; Data are presented as mean ± SD. Significance was calculated using a two-way ANOVA with multiple comparisons tests (*p < 0.05, **p < 0.01, ***p < 0.001).


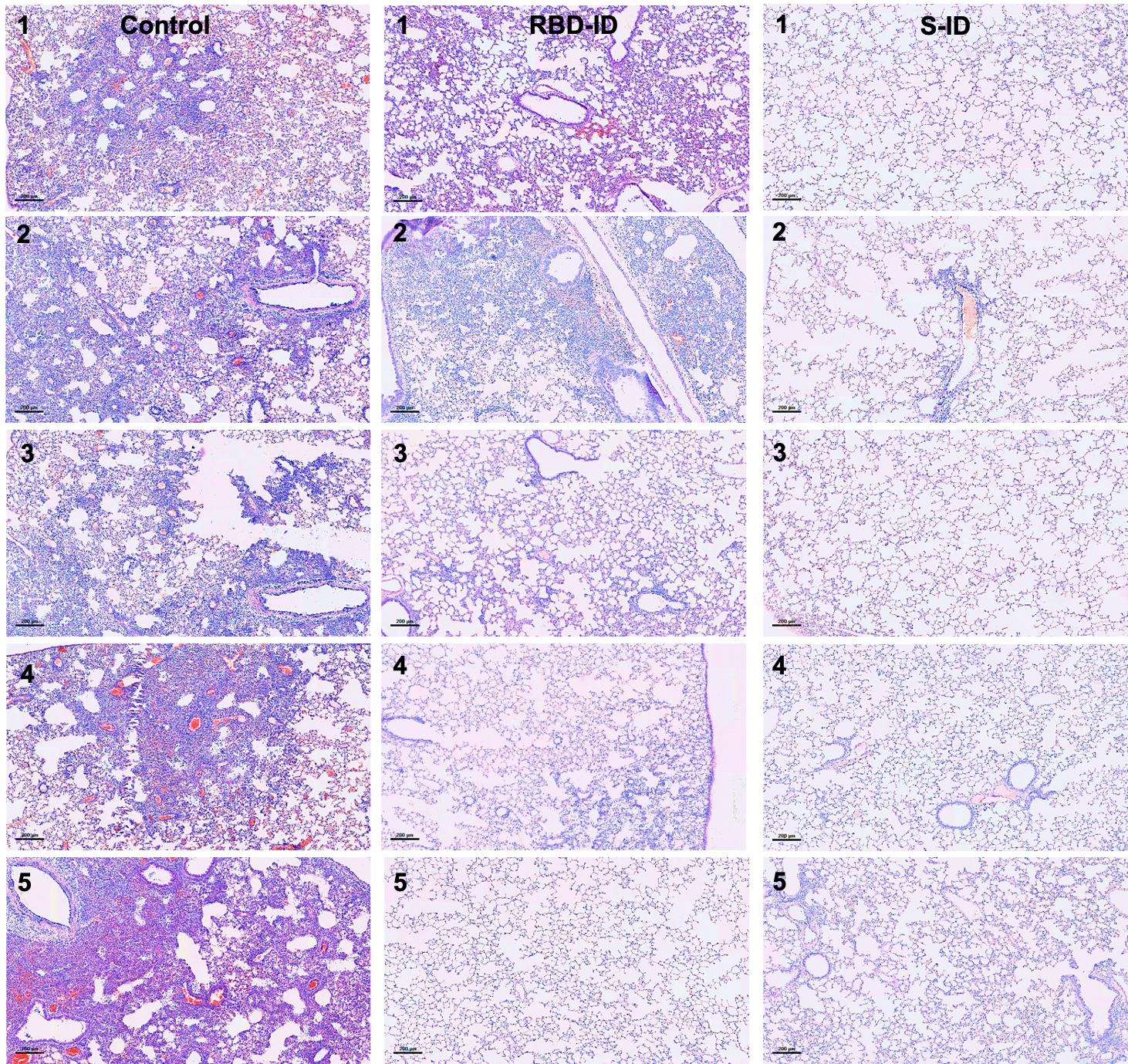


**Figure S3.** Images of hamster lung histopathological changes at 4 d.p.i.
